# A Pentaradial and Bilateral Combined Photoreception System in the Sea Cucumber is Mediated by a Blue Light‐Sensitive R‐Opsin

**DOI:** 10.1002/advs.202512095

**Published:** 2026-07-27

**Authors:** Jiasheng Huang, Chunhua Ren, Zhou Qin, Yimeng Cui, Bingbing Zhang, Peng Luo, Xuan Wang, Yaoqi Xie, Xin Zhang, Aifen Yan, Yang Zhang, Dingding Fan, Yang Liu, Wenjie Pan, Xiao Jiang, Lihong Yuan, Haipeng Qin, David M. Irwin, Chaoqun Hu, Yang Liu, Ting Chen

**Affiliations:** ^1^ State Key Laboratory of Breeding Biotechnology and Sustainable Aquaculture Laboratory of Tropical Marine Bio‐resources and Ecology Guangdong Provincial Key Laboratory of Applied Marine Biology South China Sea Institute of Oceanology Chinese Academy of Sciences Guangzhou China; ^2^ University of Chinese Academy of Sciences Beijing China; ^3^ College of Life Sciences Shaanxi Normal University Xi'an China; ^4^ School of Medicine Foshan University Foshan China; ^5^ School of Life Sciences and Biopharmaceutics Guangdong Pharmaceutical University Guangzhou China; ^6^ Agro‐Tech Extension Center of Guangdong Province Huizhou China; ^7^ Department of Laboratory Medicine and Pathobiology University of Toronto Toronto Ontario Canada

**Keywords:** light‐sensing behavior, molecular evolution, opsin, photoreceptive structure, sea cucumber

## Abstract

Despite lacking a definitive visual organ, sea cucumbers clearly demonstrate an ability to perceive light. This study identifies the anterior oral tentacles, dorsal papillae, and ventral tube feet as the light‐sensitive areas of the tropical sea cucumber *Holothuria leucospilota*. In *H. leucospilota* adults, only one active‐site‐containing opsin, specifically a rhabdomeric opsin (r‐opsin), is expressed in the light‐sensitive areas, thus providing an opportunity to characterize its photoreceptive structures through localization of this single gene. This r‐opsin is functionally confirmed to be light‐sensitive and capable of activating the Gq pathway. It exhibits a maximum absorption wavelength at 459 nm, which overlaps with the spectrum of blue light that triggers negative phototaxis responses in *H. leucospilota*, and it also mediates the responses to adjacent wavelengths. The r‐opsin‐expressing cells are distributed in the oral tentacles, papillae, and tube feet, forming distinct light‐sensing structures with specialized morphologies. These structures are interconnected by nerve cords, establishing a comprehensive photoreception system throughout the body, which integrates the diffuse photoreception of pentaradial animals and the anterior photoreception seen in most bilateral animals. This study elucidates a unique sea cucumber photoreception system, shedding light on the last previously unexplored class within Echinodermata and offers evolutionary insights into deuterostome vision.

## Graphical abstract

The sea cucumber *Holothuria leucospilota* detects blue and adjacent wavelengths of light via an r‐opsin with a maximum absorption at 459 nm. In the oral tentacles, papillae, and tube feet, distinct light‐sensing structures with specialized morphologies are formed, constituting a unique pentaradial and bilateral combined photoreception system.

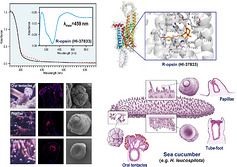

## 1 Introduction

The capacity to detect light is one of the most ancient and fundamental features of life, wherein the basic function of photoreception likely appeared before more sophisticated demands such as vision [1]. Among animals, visual organs vary significantly in anatomy, optical mechanism, size, number, position, and complexity [2]. For instance, planarians, trematodes, and polychaetes possess prototype eyes [3], nautilus, and tridacna have pinhole eyes [4], scallops and clams feature mirror eyes [5], vertebrates and cephalopods (excluding nautilus) exhibit camera eyes [6], and insects rely on compound eyes [7]. Echinoderms are the largest group of marine benthic deuterostomes, and are comprised of over 7000 extant species distributed across five classes: Crinoidea (crinoids), Ophiuroidea (brittle stars, or ophiuroids), Asteroidea (sea stars, or asteroids), Echinoidea (sea urchins, or echinoids), and Holothuroidea (sea cucumbers, or holothurians) [8]. Echinoderms were once thought to lack a distinct visual organ and have only a limited ability to sense light [9]. However, recent studies have revealed that certain echinoderms possess specialized “eyes”, such as, a whole‐body photoreceptor network with calcitic microlenses has been described in brittle stars [10, 11], ocellar and nonocellar photoreceptors were found in sea stars [12, 13], “huge compound eyes” exist in sea urchins [14], and a complex extraocular photoreception system was characterized in crinoids [15]. In addition, fossil evidence suggests that some ancient echinoderms had ossicles with microlenses that potentially function as sophisticated photosensory organs [16]. However, to date, it is unclear what type of visual organ is used by sea cucumbers.

Animals typically employ opsin as photoreceptor molecules for their visual systems [17]. Opsin is a class of G protein‐coupled receptor that covalently binds a chromophore, usually 11‐*cis*‐retinal, via a lysine to form a visual pigment that initiates photoreception due to the isomerization of retinal to its all‐*trans* form when it absorbs light [18]. Opsin families are present in Bilateria [19], Cnidaria [20], and Ctenophora [21], as well as the base of Metazoa [22]. Genome sequencing of multiple echinoderm species, including the sea urchin [23], sea star [13], brittle star [24], and crinoid [15], has enabled the identification of opsin genes [25, 26] that have been classified into the ciliary‐ (c‐), rhabdomeric‐ (r‐), and RGR/Go‐types. Phylogenetic analyses have revealed that echinoderms harbor a diverse array of opsin and opsin‐like genes, which include 7 out of the 9 major classes of bilaterian opsins [27]. Specifically, two classes, echinopsin‐A and echinopsin‐B, appear to be almost restricted to echinoderms [28]. The distribution of photoreceptor cells in the echinoderm body was inferred through the localization of opsin mRNA and proteins, which allowed speculation on the morphology and structure of their photoreceptive organs [11, 14, 15]. However, unlike vertebrates [29], the lack of functional studies on these opsins in echinoderms has left them largely uncharacterized.

**FIGURE 1:**
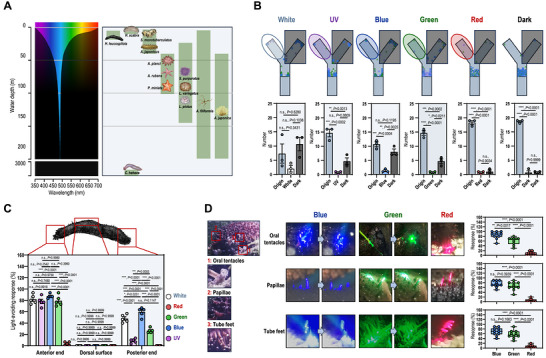
Distribution depth of echinoderms and the light‐sensing response of *H. leucospilota*. (A) The distribution depth of different species of echinoderms (*A. japonica*, *S. purpuratus*, *L. variegatus*, *L. pictus*, *A. planci*, *A. rubens*, *P. miniata*, *C. heheva*, *S. monotuberculatus*, *A. japonicus*, *H. scabra*, and *H. leucospilota*) in the ocean and the corresponding wavelengths of light at those depths. Marine spectrum at different depths was adapted from Ref. [31]. (B) The Y‐maze experiment illustrates the movement patterns of *H. leucospilota* under dark conditions or to exposure to white, red, green, blue, and UV light. The Y‐maze was divided into three sections: origin, dark, and light areas. Distribution of the final positions of sea cucumbers after 1 h across three independent trials is indicated by the purple circles, blue squares, and green triangles, respectively for each light condition. Corresponding *H. leucospilota* numbers are displayed in the bar graphs. Behavioral data are expressed as mean±SE (*n* = 3 individual experiments, each containing 20 individuals). (C) Frequency of body contractions induced by white, red, green, and blue light on the anterior end, dorsal surface, and posterior end of the sea cucumbers. Behavioral data are expressed as mean±SE (*n* = 5 individual experiments, each containing 30 individuals). (D) Appearance of *H. leucospilota* oral tentacles, papillae, and tube feet, along with their contraction responses to red, green, and blue light generated by laser pointer irradiation. Behavioral data are expressed as mean±SE (*n* = 10 from 10 individuals, each exposed to light 10 times). For statistical analysis, *P* values are calculated by one‐way ANOVA followed by Tukey's multiple comparisons test, where ^*^ *p* < 0.05, ^**^ *p* < 0.01, ^***^ *p* < 0.001, ^****^ *p* < 0.0001. Detailed contraction processes are presented in the Videos S1–S4.

Like many echinoderms, sea cucumbers from the order Aspidochirota are marine benthic species that primarily inhabit shallow coastal waters and coral reefs [30], where blue light is abundant [31], although some species are found in the completely dark deep‐sea (Figure 1A) [32]. Sea cucumbers exhibit a pentaradial symmetry that is similar to other echinoderms; however, fossil evidence indicates that some echinoderm ancestors displayed spiral, asymmetric, or bilateral symmetries [33, 34]. Unlike other echinoderms such as sea urchins, sea stars, brittle stars, and crinoids, most sea cucumbers have reacquired anterior and posterior ends and exhibit locomotion with forward movement [35]. Sea cucumbers have been reported to exhibit either phototactic [36] or photophobic behaviors [37], depending on specific conditions. Light cycles and intensities may affect the daily activity rhythm of *Apostichopus japonicus* [38], and significantly impact their growth, feeding, digestion efficiency, larval development, and juvenile growth [39, 40]. In the synaptid holothurian, *Opheodesoma spectabilis*, an ocellus‐like structure has been identified on the oral tentacles [41], but the overall photoreception system in these species remains unclear. *Holothuria leucospilota* is an ecologically important tropical sea cucumber that is widely distributed across the Western Pacific and Indian Oceans [42], and its chromosome‐level genome assembly has been reported [43]. Here, we present the unique photoreception system of *H. leucospilota*, which is localized within the oral tentacles, papillae, and tube feet, and is mediated by a blue light‐sensitive r‐opsin that activates the Gq pathway. This photoreception system combines the characteristics of diffuse photoreception found in pentaradial animals and anterior photoreception observed in most bilateral animals. The elucidation of the light‐sensing mechanism in Holothuroidea offers novel perspectives on the diversity and evolution of the visual system.

## 2 Results

### 2.1 The Light‐Sensing Response and Photosensitive Parts of *H. leucospilota*

To characterize light avoidance by the sea cucumber, a behavioral experiment was conducted using a Y‐maze assay with different artificial light sources, including a full spectrum white light source (400–830 nm) and four monochromatic light‐emitting diodes (LEDs) emitting red (620–630 nm), green (510–520 nm), blue (465–470 nm), and ultraviolet (UV) (365–375 nm) light. *H. leucospilota* displayed a strong negative phototaxis response to white and blue light, weak negative phototaxis towards green and UV light, and no reaction to red light (Figure 1B and Video S1).

To identify the site of the light‐sensing organ in the sea cucumber, the anterior, posterior, and dorsal parts of *H. leucospilota* were illuminated with lasers with different wavelengths of light, and the response of the animal, in terms of body contractions to avoid light exposure, was observed. The results of this experiment showed that white, blue, green, and UV light exposure on the anterior and posterior parts triggered significant contraction responses, with the most pronounced reactions occurring with white and blue light, while red light did not elicit any response (Figure 1C). Furthermore, illuminating the dorsal parts of the sea cucumber with any type of light failed to induce any contraction response (Figure 1C).

Through laser pointer stimulation, the light sensors of *H. leucospilota* were identified to be located in the anterior oral tentacles, dorsal papillae, and abdominal tube feet, with a specific distribution of photosensitive sites at the tips of the oral tentacles and papillae, and the suckers of the tube feet (Figure 1D). The strongest contraction responses, where head retraction was observed, were observed for the oral tentacles, while the papillae and tube feet displayed partial contractions (Figure 1D and Videos S2–S4). However, all three structures exhibited responses only to blue and green laser light, whereas no response was observed to red laser light (Figure 1D and Videos S2–S4).

### 2.2 Genome‐Wide Identification and Phylogenetic Analysis of Opsin Genes From *H. leucospilota*

**FIGURE 2:**
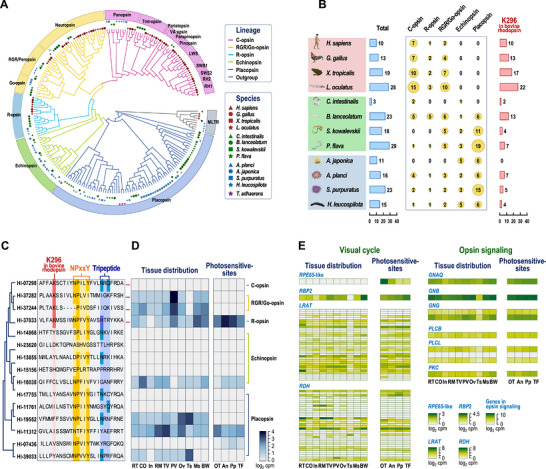
Identification and transcriptional analysis of opsins in *H. leucospilota*. (A) Phylogenetic analysis of opsins in 12 typical deuterostome species (*H. sapiens*, *G. gallus*, *X. tropicalis*, *L. oculatus*, *C. intestinalis*, *B. lanceolatum*, *S. kowalevskii*, *P. flava*, *A. planci*, *A. japonica*, *S. purpuratus*, and *H. leucospilota*) and a placozoan (*T. adhaerens*) species. The full phylogenetic tree is shown in Figure S1. (B) Numbers of opsin genes in the 12 typical deuterostome species. (C) Characteristics of conserved sequences in the seventh transmembrane domain of *H. leucospilota* opsins. (D) Heatmap illustrating transcript expression levels of *H. leucospilota* opsins in different tissues including the respiratory tree (RT), Cuvierian organ (CO), intestine (In), rete mirabile (RM), transverse vessel (TV), Polian vesicle (PV), ovary (Ov), testis (Ts), muscle (Ms) and body wall (BW), and photosensitive‐sites including the oral tentacles (OT), anus (An), papillae (Pp), and tube feet (TF). (E) Heatmap illustrating transcript expression levels of genes related to the visual cycle and opsin signaling in different tissues and photosensitive‐sites, as described above. *RPE65*‐like, retinal pigment epithelium 65 kDa protein like; *RBP2*, retinol‐binding protein 2; *LRAT*, lecithin retinol acyltransferase; *RDH*, retinol dehydrogenase; *GNAQ*, G protein subunit alpha q; *GNB*, G protein subunit beta; *GNG*, G protein subunit gamma; *PLCB*, 1‐phosphatidylinositol 4,5‐bisphosphate phosphodiesterase beta; *PLCL*, inactive phospholipase C‐like protein; *PKC*, protein kinase C.

Through genome‐wide screening, a total of 208 opsin genes were identified among 12 representative deuterostome species. These opsins were classified into 5 major classes, and 17 subclasses, based on their phylogenetic relationships: c‐opsin (rhodopsin [RH1], rhodopsin‐like [RH2], short‐wavelength sensitive 1 opsin [SWS1], short‐wavelength sensitive 2 opsin [SWS2], long‐wavelength sensitive opsin [LWS], pinopsin, parapinopsin, parietopsin, vertebrate ancient [VA] opsin, teleost multiple tissue [Tmt]‐opsin, and panopsin), r‐opsin, RGR/Go‐opsin (neuropsin, peropsin, and Go‐opsin), echinopsin that is absent in vertebrates, and placopsin that is similar to Placozoan opsins (Figure 2A and Figure S1 and Table S1). Compared to vertebrates, which exhibit a higher prevalence of classical c‐opsin, r‐opsin, and RGR/Go‐opsin, ambulacrarians (hemichordates and echinoderms) show an increased representation of echinopsin and placopsin genes (Figure 2B). *H. leucospilota* possesses 15 opsins, with the non‐classical types predominating, which include 1 c‐opsin, 1 r‐opsin, 2 RGR/Go‐opsins, 5 echinopsins, and 6 placopsins (Figure 2B).

The lysine residue corresponding to K296 in the bovine rhodopsin sequence serves as a crucial chromophore binding site, which allows the formation of light‐sensitive visual pigment. Within the 15 *H. leucospilota* opsins, 4 sequences contain this conserved lysine residue (Figure S2). Among these, r‐opsin (Hl‐37833) and c‐opsin (Hl‐07298) also exhibit the conserved NPxxY motifs involved in G‐protein activation. Notably, Hl‐07298 possesses the complete c‐opsin‐characteristic NxQ motif, whereas Hl‐37833 retains only a partial version of the r‐opsin‐characteristic HxK motif (Figure 2C and Figure S2).

Transcriptomic analysis revealed that of the 15 opsin genes in the *H. leucospilota* genome, only eight are expressed in adult tissues, including r‐opsin (Hl‐37833) and two RGR/Go‐opsins (Hl‐37282 and Hl‐37244), whereas no evidence for c‐opsin (Hl‐07298) expression was found (Figure 2D and Tables S2 and S3). Although expression of r‐opsin and RGR/Go‐opsins was detected in the body wall, only r‐opsin (Hl‐37833) was expressed in the photosensitive oral tentacles, anus, papillae, and tube feet (Figure 2D), which was confirmed using a highly sensitive RT‐PCR assay (Figure S3). These findings strongly suggest that r‐opsin (Hl‐37833) serves as the primary opsin mediating photoreception in *H. leucospilota*. Furthermore, the expression pattern analysis showed that the homolog gene of *RPE65*, a key retinol isomerase involved in the vertebrate visual cycle [44, 45], was specifically expressed at the photosensitive sites (Figure 2E and Figure S4 and Tables S2 and S3). In contrast, other genes in the visual cycle, such as *RBP2*, *LRAT*, and *RDH*, along with most genes associated with opsin signaling cascades, exhibited ubiquitous expression (Figure 2E and Figure S4 and Tables S2 and S3).

### 2.3 Spectral Tuning and Structural Analysis of *H. leucospilota* R‐Opsin

**FIGURE 3:**
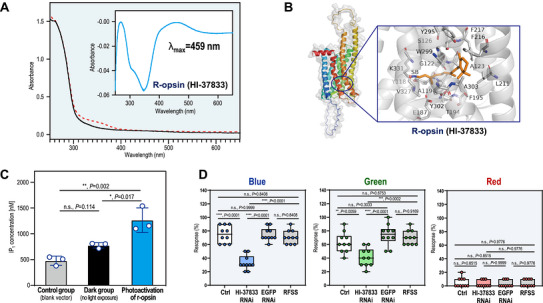
Functional characterizations of *H. leucospilota* r‑opsin (Hl‑37833). (A) In vitro assays of the *H. leucospilota* r‐opsin (Hl‐37833). The black line represents the absorption spectrum in the dark state, and the red line represents the spectrum after light exposure in the presence of hydroxylamine. The inset (blue line) is the difference spectrum calculated from the dark and light‐exposed spectra, with λ_max_ indicated. (B) Molecular docking model of *H. leucospilota* r‐opsin (Hl‐37833) binding with 11‐*cis*‐retinal by a Schiff base (SB) linkage to the K331. The putative counterion E187, as well as amino acid residues within a 4 Å distance of the ligand, are indicated. (C) IP_1_ assay for *H. leucospilota* r‐opsin (Hl‐37833) expressed in cultured cells. Gray, black, and blue bars represent the control (blank pcDNA3.1 vector), no light (dark), and blue‐light‐exposure groups. Three independent experimental replicates were performed, each with three technical repeats. (D) Effects of RNAi targeting r‑opsin (Hl‑37833) on the contraction responses of *H. leucospilota* oral tentacles upon stimulation with blue (450 nm), green (532 nm), and red (650 nm) laser pointers. Behavioral data are expressed as mean±SE (*n* = 10 from 10 individuals, each exposed to light 10 times). For statistical analysis, *p* values are calculated by one‐way ANOVA followed by Tukey's multiple comparisons test, where ^*^ *p* < 0.05, ^**^ *p* < 0.01, ^***^ *p* < 0.001, ^****^ *p* < 0.0001.

An in vitro spectral sensitivity assay was performed on r‐opsin (Hl‐37833), which is highly expressed in the photosensitive sites of *H. leucospilota*. The regenerated visual pigment was found to be light‐sensitive, with a wavelength of maximum absorption (λ_max_) of 459 nm (Figure 3A and Table S4). The λ_max_ measured after light exposure alone was similar (457 nm) (Figure S5 and Table S4). This value closely aligns with the blue light (465–470 nm) that induces strong negative phototaxis in *H. leucospilota* (Figure 1B). The inferred structural model of *H. leucospilota* r‐opsin (Hl‐37833) further supports that 11‐*cis*‐retinal could bind lysine at site 331 (homologous to K296 in bovine rhodopsin) covalently by a Schiff base linkage [46]. In addition, E187 is predicted to be the counterion, corresponding to the counterion residues identified in squid and bovine rhodopsins [47, 48, 49] (Figure 3B).

### 2.4 Signal Transduction

To evaluate whether *H. leucospilota* r‐opsin activates the Gq signaling pathway, we measured the accumulation of inositol monophosphate (IP_1_). Photoactivation of r‐opsin (Hl‐37833) significantly increased IP_1_ levels compared with both the control group (blank vector) and the dark group (no light exposure). No significant difference was observed between the control and dark groups (Figure 3C and Figure S6). These results indicate that *H. leucospilota* r‐opsin can activate the Gq pathway upon blue‐light stimulation.

### 2.5 Mediation of *H. leucospilota* Light‐Sensing Response by R‐Opsin

RNA interference (RNAi) significantly reduced the mRNA expression of r‐opsin (Hl‐37833) in the photosensitive tissues (Figure S7). Silencing of the r‐opsin (Hl‐37833) transcript markedly diminished the contraction responses of *H. leucospilota* oral tentacles to blue and green laser light. In contrast, neither the EGFP RNAi nor the RNase‐free saline solution (RFSS) injection controls had any effect on the light‐induced responses. Furthermore, no responses were observed to red laser illumination (Figure 3D). These results demonstrate that the photoresponses to both blue and green light in *H. leucospilota* are primarily mediated by r‐opsin (Hl‐37833).

### 2.6 Comparative Analysis of Opsins From *H. leucospilota* and Other Echinoderms

**FIGURE 4:**
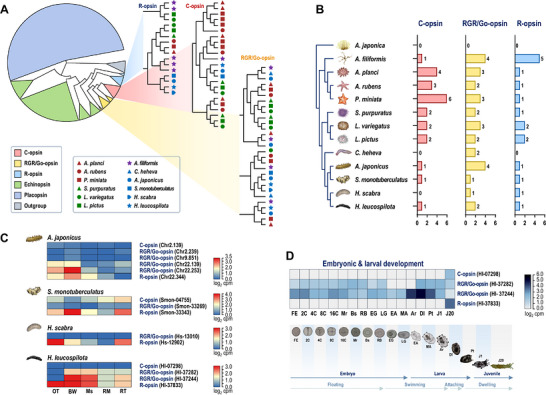
Comparative genomic and expression analysis of opsins in the sea cucumber. (A) Phylogenetic analysis of opsins from 12 echinoderm species with high‐quality available genomic data (*A. filiformis*, *A. planci*, *A. rubens*, *P. miniata*, *S. purpuratus*, *L. variegatus*, *L. pictus*, *C. heheva*, *A. japonicus*, *S. monotuberculatus*, *H. scabra*, and *H. leucospilota*). The full phylogenetic tree is shown in Figure S8. (B) Numbers of opsin genes in these 13 echinoderm species (the above 12 species and *A. japonica*). (C) Heatmap illustrating transcript expression levels of opsins in different tissues of 4 holothurian species (*A. japonicus*, *S. monotuberculatus*, *H. scabra*, and *H. leucospilota*). (D) Heatmap illustrating transcript expression levels of *H. leucospilota* opsins at different stages of embryonic and larval development. The morphologies and characteristics corresponding to each developmental stage are shown below.

Opsin genes were identified in 13 echinoderm species with high‐quality genome assemblies (Table S1). Those containing chromophore‐binding sites were classified into the classical c‐opsin, r‐opsin, and RGR/Go‐opsin categories (Figure 4A and Figure S8). Interestingly, crinoids appear to lack opsin genes entirely, whereas ophiuroids possess the highest number of opsin genes, followed by asteroids and echinoids, while holothurians have comparatively fewer opsins (Figure 4B). All shallow‐water sea cucumbers possess one r‐opsin gene (Figure 4B), and these genes are phylogenetically homologous (Figure 4A). Surprisingly, the deep‐sea sea cucumber *Chiridota heheva*, which inhabits a light‐deprived deep‐sea environment, harbors two RGR/Go‐opsin genes despite lacking an r‐opsin gene, one of which contains a chromophore‐binding site (Figure 4B and Figure S8).

Opsin gene expression was also detected in several sea cucumber species, including *A. japonicus*, *Stichopus monotuberculatus*, and *Holothuria scabra*, in addition to *H. leucospilota*. Across these species, r‐opsins exhibited higher expression levels than c‐opsins (Figure 4C and Table S5). R‐opsins were expressed not only in photosensitive tissues such as the oral tentacles and body wall but also in internal tissues (Figure 4C and Table S5), suggesting that they may have additional physiological roles beyond photoreception in sea cucumbers.

### 2.7 Opsin Expression in *H. leucospilota* During Embryonic and Larval Development

Throughout the embryonic and larval developmental stages of *H. leucospilota*, the RGR/Go‐opsins (Hl‐37282 and Hl‐37244) are consistently expressed. Conversely, expression of c‐opsin (Hl‐07298) and r‐opsin (Hl‐37833) is absent until the juvenile stage, when individuals reach a length of approximately 20 mm (Figure 4D and Table S6). In adults, c‐opsin (Hl‐07298) expression is no longer detectable, whereas r‐opsin (Hl‐37833) remains expressed in photoreceptive tissues (Figure 2D).

### 2.8 Photoreceptors and Connections to the Neural System of *H. leucospilota*

**FIGURE 5:**
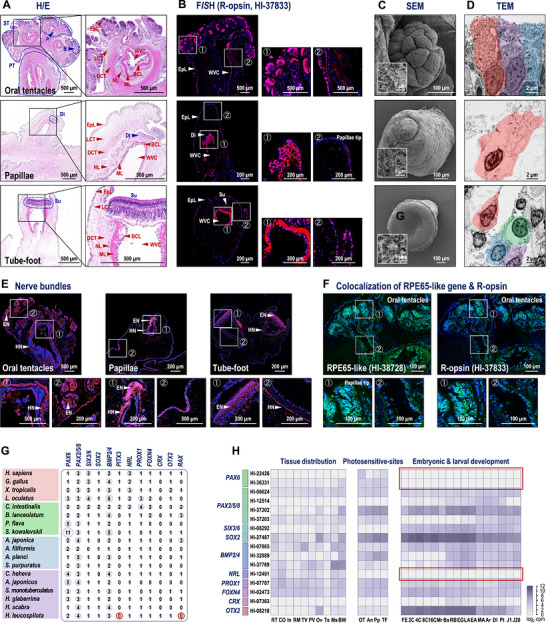
Histology, morphology, and opsin and eye development‐related gene expression of photoreceptors in the oral tentacles, papillae, and tube feet of *H. leucospilota*. (A) H/E staining for histology of the oral tentacles, papillae, and tube feet. High‐resolution images are provided in Figures S9 and S10. (B) F*IS*H for r‐opsin (Hl‐37833) mRNA expression (red), with cell nuclei stained with DAPI (blue). High‐resolution and negative‐control images are provided in Figure S11. (C) SEM for surface ultrastructure of photoreceptors. The inset shows ultrastructural details at high magnification. High‐resolution images are also provided in Figure S12. (D) TEM for internal ultrastructure of photoreceptors. High‐resolution images are provided in Figure S13. (E) Anti‐*β*‐tubulin staining for nerve bundles (red), with cell nuclei stained with DAPI (blue). High‐resolution and negative‐control images are provided in Figure S14. (F) F*IS*H for colocalization of *RPE65*‐like gene (Hl‐38728) and r‐opsin (Hl‐37833) mRNA expression (green) in the photosensitive oral tentacles, with cell nuclei stained with DAPI (blue). High‐resolution and negative‐control images are provided in Figure S15. PT, petiole tentacles; ST, scutiform tentacles; P, papillae; B, buds; Di, disc; Su, sucker; EpL, epithelial layer; LCT, loose connective tissue; DCT, dense connective tissue; NL, nerve layer; ML, muscle layer; BCL, body cavity lining; WVC, water vascular cavity; EN, epineural nerve plexus; HN, hyponeural nerve plexus. (G) Numbers of genes related to eye development in 8 typical deuterostome (*H. sapiens*, *G. gallus*, *X. tropicalis*, *L. oculatus*, *C. intestinalis*, *B. lanceolatum*, *S. kowalevskii*, and *P. flava*) and 9 echinoderm (*A. japonica*, *A. filiformis*, *A. planci*, *S. purpuratus*, *C. heheva*, *A. japonicus*, *S. monotuberculatus*, *H. scabra*, and *H. leucospilota*) species. *PAX6*, paired box protein‐6; *PAX2*/*5*/*8*, paired box protein‐2/5/8; *SIX3*/*6*, homeobox protein‐3/6; *SOX2*, transcription factor *SOX‐2*; *BMP2*/*4*, bone morphogenetic protein‐2/4; *PITX3*, pituitary homeobox‐3; *NRL*, neural retina‐specific leucine zipper protein; *PROX1*, prospero homeobox protein‐1; *FOXN4*, forkhead box protein‐N4; *CRX*, cone‐rod homeobox; *OTX2*, orthodenticle homeobox‐2; *RAX*, retinal homeobox protein. (H) Heatmap illustrating transcript expression levels of genes related to eye development in different tissues, photosensitive‐sites, and at embryonic and larval developmental stages.

The oral tentacles, papillae, and tube feet of *H. leucospilota* are specialized structures extended from its water vascular system. These structures comprise multiple tissue layers arranged around the water vascular cavity, and include the epithelial layer, loose connective tissue, dense connective tissue, nervous layer, muscular layer, and body cavity lining (Figure 5A and Figure S9). The oral tentacles are further differentiated into the petiole tentacles and the scutiform tentacles, which are characterized by mastoid processes (Figure 5A and Figure S10). Papillae and tube feet, on the other hand, are distinguished by the presence of an adhesive disc and a sucker at their tips, respectively (Figure 5A and Figure S10).

Fluorescence in situ hybridization (F*IS*H) was employed to identify cells expressing r‐opsin (Hl‐37833) mRNA, thus identifying the putative photoreceptor cells (PRCs). In the oral tentacles, PRCs were found on the surface at the terminus of the scutiform tentacles. In contrast, within the papillae and tube feet, the major PRCs were not located on the surface but rather beneath the disc of the papillae and the sucker of the tube feet, respectively, surrounding the upper portion of the water vascular cavity (Figure 5B and Figure S11). Additionally, weak signals for Hl‐37833 were also observed in the epithelial layer and connective tissue. By comparison with the negative controls, the signals in the epithelial layer were confirmed as specific, whereas those in the connective tissue were considered nonspecific and were therefore excluded (Figure S11).

Scanning electron microscopy (SEM) revealed distinct morphological differences among the photoreceptive structures of *H. leucospilota*. The oral tentacles featured multiple mastoid projections at their termini, while the papillae exhibited a single protrusion at their apex, and the tube feet possessed a concave sucker (Figure 5C). Under high magnification, numerous microvilli were observed on these structures (Figure 5C and Figure S12). Transmission electron microscopy (TEM) further revealed that these photoreceptor cells, characterized by their microvilli, were located on the surface of the oral tentacles and within the concave regions at the tips of the papillae and tube feet (Figure 5D and Figure S13).

Immunostaining using antibodies against acetylated tubulin revealed that extensive nerve fiber networks were distributed within the epithelial layer of the oral tentacles, as well as the disc of the papillae and the sucker of the tube feet (Figure 5E and Figure S14). These nerve fibers merged into bundles within the outer neural layer adjacent to the water vascular cavity with the epineural nerve plexus and hyponeural nerve plexus and extended downwards to either the circumoral nerve ring or the radial nerve cords of the nervous system (Figure 5E and Figure S14). Furthermore, F*IS*H further revealed that r‐opsin (Hl‐37833) and the *RPE65*‐like gene (Hl‐38728) exhibit closely overlapping expression patterns in the oral tentacles of *H. leucospilota* (Figure 5F and Figure S15).

### 2.9 Comparative Genomic and Expression Analysis of Eye Development‐Related Genes in *H. leucospilota*

Comparative analysis of genes associated with eye development revealed that the *H. leucospilota* genome contains 2, 4, and 3 copies of the *PAX6*, *PAX2*/*5*/*8*, and *BMP2*/*4* genes, respectively (Figure 5G and Table S7). Additionally, a single copy of the *SIX3*/*6*, *SOX2*, *NRL*, *PROX1*, *FOXN4*, *CRX*, and *OTX2* were identified in the *H. leucospilota* genome, whereas *PITX3* and *RAX* genes were notably absent (Figure 5G and Table S7). Among deuterostomes, the loss of *PITX3* is a defining feature of echinoderms and other non‐chordate groups, distinguishing them from vertebrates (Figure 5G and Table S7).

Transcriptomic analysis also demonstrated stage‐specific gene expression patterns, during embryonic and larval development, of eye‐development genes in *H. leucospilota*. Neither *NRL*‐like nor the two *PAX6*‐like genes were expressed during early development (Table S6), but became active in various tissues of the adult (Table S2), with *PAX6*‐like genes showing the highest levels of expression at photosensitive sites (Figure 5H and Table S3). In contrast, *PAX2*/*2A*‐like, *CRX*‐like, and *OTX2*‐like genes exhibited elevated expression during larval stages (Table S6), which then decreased in adult tissues (Figure 5H and Table S2).

## 3 Discussion

Opsins play crucial roles in various biological processes of animals, particularly in vision and non‐visual photoreception [2]. They mediate a wide range of light‐dependent behaviors, including movement, feeding, mating, and predator avoidance, which are vital for survival and reproduction [50]. Despite echinoderms having relatively limited mobility [51], the total opsin repertoires of their genomes are comparable to those of more motile phyla (Figure 2B). However, most of the opsin genes found in echinoderms belong to the echinopsin and placopsin families [22, 28], while those with chromophore‐binding sites (c‐opsin, r‐opsin, and RGR/Go‐opsin) have a significantly reduced number of genes (Figures 2B and 4B). The presence of multiple opsin genes has been documented across various echinoderm classes, although many of the identified sequences are incomplete [13, 15, 23, 24, 52]. Unlike mollusks, where the diversity and abundance of opsins are independent of the complexity of the light‐sensing organs [53], echinoderm opsin diversity correlates strongly with motility, where brittle stars demonstrate the highest overall movement speed, followed by sea stars, while sea cucumbers and sea urchins exhibit comparable speeds, and crinoids are sessile [51, 54, 55, 56]. Specifically, brittle stars possess more opsins than asteroids and echinoids [24], which in turn have more than holothurians, and finally, crinoids (Figure 4B and Table S1). Although isomerization of 11‐*cis*‐retinal chromophore to the all‐*trans* conformation is essential for cellular signaling in photoreception, certain opsins with mutations at the chromophore‐binding site lack light sensitivity but still retain the ability to activate signal transduction pathways [57]. In the absence of chromophore binding, some opsins function in thermosensation [58], mechanoreception [59], or chemosensation [60]. Echinoderm‐specific opsins, such as echinopsins and placopsins, may have evolved to support these non‐light‐detection functions.

In *H. leucospilota*, four opsins with chromophore‐binding sites were identified: one c‐opsin, one r‐opsin (Hl‐37833), and two RGR/Go‐opsins (Figure 2C). Among these, only the r‐opsin (Hl‐37833) is expressed in light‐sensitive regions, such as the oral tentacles, anus, papillae, and tube feet (Figure 2D). Therefore, adult *H. leucospilota* may rely primarily on this single r‐opsin for photoreception, and its light sensitivity is confirmed by a spectral tuning experiment (Figure 3A). RNAi‐mediated knockdown of r‐opsin (Hl‐37833) expression suppressed the response of *H. leucospilota* to blue light and also reduced its response to green light (Figure 3D), suggesting that photoresponse to both wavelengths is mediated by Hl‐37833. During embryonic and larval development, RGR/Go‐opsins appear to participate in light detection, as suggested by their expression profiles (Figure 4D). As echinoderms transition from planktonic swimming larvae to benthic crawling adults, their opsin genes may acquire stage‐specific functions to meet the distinct locomotion demands of each phase. Sea cucumber auricularia and doliolaria larvae have ciliary bands like those in sea urchin larvae, where ciliary photoreceptors employ Go‐opsin to mediate light‐dependent phototaxis swimming and pyloric opening [61, 62]. The previously absent c‐opsin and r‐opsin in *H. leucospilota* are first expressed starting from the juvenile stage as they adopt a benthic motility style (Figure 4D), paralleling observations in sea urchin juveniles, which also begin expressing c‐opsin and r‐opsin while maintaining reduced levels of Go‐opsin [63, 64]. However, the functions of these opsins remain unclear in animals, as RGR/Go‐opsins interact with a G‐protein distinct from those involved in the c‐opsin and r‐opsin transduction cascades [65]. An ontogenetic shift in opsin usage between larval and adult stages is a well‐documented phenomenon in other marine animals, such as tunicates [66], annelids [67], and teleosts [68].

Adult individuals of *H. leucospilota* exhibit strong negative phototaxis in response to blue light, contracting their bodies to minimize exposure (Figure 1B). Comparable avoidance behaviors are observed under green and UV light, which are adjacent wavelengths (Figure 1C). Correspondingly, the functional r‐opsin (Hl‐37833) of adult *H. leucospilota* exhibits a λ_max_ at 459 nm (Figure 3A) and likely serves as a blue‐light–sensitive pigment. Although no clear evidence of chromophore release was observed in the absence of hydroxylamine (Figure S5), the λ_max_ value measured after light exposure was similar. Furthermore, the observed increase in IP_1_ production following light exposure indicates that *H. leucospilota* r‐opsin is capable of activating the Gq signaling pathway (Figure 3C), supporting that this sea cucumber opsin can drive G protein‐dependent signaling pathways in a light‐dependent manner. However, whether it can also activate other downstream pathways, such as the previously reported Gs, Go, and Gi [69, 70], remains unclear.

The homologous r‐opsin genes are ubiquitously expressed in the light‐sensitive areas of various shallow‐water sea cucumbers (Figure 4C), indicating that photoreception mediated by r‐opsin represents a conserved mechanism across holothurians. Likewise, blue starfish *Linckia laevigata* [71], crown‐of‐thorns starfish *Acanthaster planci* [72], and crinoid *Antedon bifida* [15] have been found to be most sensitive to blue light at approximately 450, 470, and 463 nm, respectively, based on behavioral experiments. This trend suggests that sensitivity to blue wavelengths may be a common adaptive trait among echinoderms. Since light perception in marine habitats is challenged due to reduced light intensity and a narrower, blueshifted spectrum compared to terrestrial environments, blue‐light sensitivity could optimize their photoreception [73]. Most marine invertebrates lack complex color vision, possessing only monochromacy or dichromacy [74]. Similarly, marine vertebrates such as cetaceans and sharks exhibit a spectral blueshift in their visual pigments compared to terrestrial relatives [75].

**FIGURE 6:**
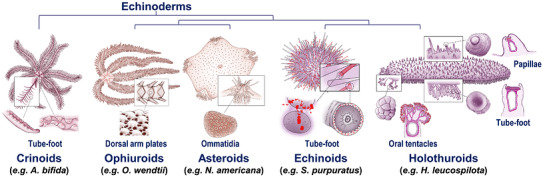
The photoreception systems of Holothuroids (e.g.*, H. leucospilota*) and Crinoids (e.g.*, A. bifida*), Ophiuroids (e.g., *O. wendtii*), Asteroids (e.g., *N. americana*), Echinoids (e.g., *S. purpuratus*) in Echinodermata.

In the adult *H. leucospilota*, only one functional r‐opsin (Hl‐37833) is expressed in its light‐sensitive areas (Figure 2C). This r‐opsin is capable of mediating photoresponses to adjacent wavelengths (Figure 3D), thus offering an opportunity to characterize the corresponding photoreceptive structures through localization of this single gene. Within the oral tentacles, papillae, and tube feet, r‐opsin‐expressing photoreceptor cells form specialized light‐sensing structures (Figure 4B). Correspondingly, a complete set of visual cycle‐related genes is exclusively expressed at these sites (Figure 2F). In addition, the *RPE65*‐like gene is expressed in areas of the oral tentacles that largely overlap with r‐opsin expression (Figure 5F), suggesting a potential functional association between this invertebrate RPE65‐like protein and r‐opsin, similar to the visual cycle reported in ascidians and cephalopods [76, 77]. However, whether this gene participates in the visual cycle requires further investigation [78, 79]. However, the absence of key eye development regulators *PITX3* and *RAX* (Figure 5G), combined with the silencing of *NRL* and *PAX6* during development (Figure 5H), has prevented the formation of fully differentiated visual organs in sea cucumbers. Despite lacking conventional eyes, *H. leucospilota* possesses a unique photoreception system, integrating multiple sensory structures across the body that are interconnected by nerve fibers (Figure 5B,E). These nerve fibers extend from the photoreceptor cells in the oral tentacles towards the circumoral nerve ring, while those in the papillae and tube feet project toward the radial nerve cords [80]. The diffuse photoreception system, comprising different types of photoreceptor cells spread throughout the body, is a typical characteristic of echinoderms, which exhibit pentaradial symmetry. This feature has also been observed in other echinoderms (Figure 6), including sea urchins that rely on photoreceptor cells localized on the disk and basal regions of their tube feet [14], sea stars that exhibit both compound eyes at the tips of their arms and dermal photoreception across their bodies [13, 71, 72], crinoids that possess photoreceptor cells on the sensory papillae of their tube feet and within the ectoneural basiepithelial and hyponeural nerve plexus [15], as well as brittle stars that feature an extensive whole‐body photoreceptor network [11].

The evolution of photoreception and vision proceeds through four stages: non‐directional photoreception, directional photoreception, low‐resolution vision, and high‐resolution vision [81]. In bilateral animals, visual organs are typically concentrated in the anterior region to optimize navigation and environmental awareness [81]. Conversely, non‐directional photoreception in animals not only controls movement but also supports additional physiological functions [82], leading to reduced cephalic eyes and the evolution of new photoreceptors on their bodies in some lineages [81]. In *H. leucospilota*, photoreception is functionally divided into two distinct systems: directional photoreception localized in the anterior oral tentacles and non‐directional photoreception distributed throughout the papillae and tube feet (Figure 6), mediating negative phototaxis movements and light avoidance contractions (Figure 1C,D). This dual arrangement reflects a remarkable integration of two evolutionary strategies: the diffuse photoreception typical of echinoderms with pentaradial symmetry and the anteriorly concentrated photoreception characteristic of their bilaterally symmetric ancestors. Thus, *H. leucospilota* exhibits a hybrid photoreception system that incorporates both pentaradial and bilateral traits, highlighting evolutionary transitions between the body plans of echinoderms (Figure 6).

The two photoreception mechanisms of *H. leucospilota* are speculated to perform distinct roles. The anterior oral tentacles, which are most exposed to external light, exhibit the strongest phototaxis responses (Figure 1C), indicating a potential role in mediating forward movement and exploratory behaviors. In contrast, dorsal papillae show responses that are less immediate (Figure 1C,D), suggesting an involvement in rhythmic environmental sensing, possibly linked to diel activity regulation or long‐term light exposure monitoring [38]. Meanwhile, ventral tube feet, which remain in direct contact with the substrate, are more likely involved in substrate recognition and microhabitat selection, rather than in detecting rapid changes in illumination [37]. The subsurface positioning of photoreceptor cells within the sucker region (Figure 5A,B) further supports the idea that tube feet are adapted for assessing ambient environmental light. In shallow coastal environments, strong blue light penetration may serve as a reliable cue for distinguishing between exposed and sheltered microhabitats [30], allowing sea cucumbers to adjust their hiding or burrowing behaviors accordingly.

Despite the unique body plans, echinoderms remain evolutionarily conserved among deuterostomes, with their pentaradial symmetry evolved from bilaterally symmetrical ancestors [33, 83]. Recent studies suggest echinoderms are mostly “head‐like animals”, with each midline ray representing anterior territory while the outermost part shows posterior identity [84]. Under this model, we propose that ancestral photoreceptor cells and neural networks were retained during echinoderm evolution. Following the “head” expansion, anterior‐concentrated photoreceptor cells spread posteriorly throughout the body, enabling widespread whole‐organism light detection. In sea cucumbers, photoreceptors in the papillae and tube feet confirm these structures as sensory appendages (Figure 6). However, when sea cucumbers re‐evolved forward movement, photoreceptors underwent a secondary reaggregation at the anterior oral tentacles, akin to the cephalic light sensory structures of bilateral deuterostomes (Figure 6).

Taken as a whole, this study reveals a previously unrecognized photoreception system in the sea cucumber *H. leucospilota*, which integrates both pentaradial and bilateral characteristics. This system consists of light‐sensitive cells in anterior oral tentacles, as well as in the papillae and tube feet that are distributed across the body, all of which are interconnected by nerve cords to facilitate photoreceptive signaling. Central to this system is an r‐opsin with peak sensitivity at 459 nm, enabling the detection of blue‐light and lights with adjacent wavelengths, and triggering a range of light‐induced contraction responses and avoidance behaviors. These findings reveal sophisticated sensory adaptations in sea cucumbers, demonstrating their retention of diffuse photoreception alongside re‐evolved anterior photoreceptive structures associated with directional locomotion.

## 4 Experimental Section

### 4.1 Animals and Reagents

Adult *H. leucospilota* specimens of mixed sex, measuring around 20 cm in length and weighing approximately 400 g, were collected from Daya Bay (112°20′E, 16°50′N) in Shenzhen City, China. The sea cucumbers were then acclimated for 2 weeks in cement ponds filled with seawater at 30‰ salinity and pH 8.0, under standard photoperiod conditions. Behavioral experiments were carried out either in a Y‐maze or in glass tanks. Tissue samples were obtained from the sea cucumbers after they were anesthetized by immersion in a seawater solution containing 5% MgCl_2_. All animals were handled in accordance with the research guidelines set up by the research ethics committee for animal experiments at the South China Sea Institute of Oceanology, Chinese Academy of Sciences, with the approval number of 2022‐0011. Information regarding the reagents and oligonucleotides used in this study is shown in Table S8.

### 4.2 Light‐Sensing Behaviors

The light‐sensing behaviors of sea cucumbers were examined in a Y‐maze (main channel 1.0 m long, channel width of 0.25 m, each arm 0.6 m long) with various light sources. The light sources utilized included a full‐wavelength white light (400–830 nm), as well as red (620–630 nm), green (510–520 nm), blue (465–470 nm), and ultraviolet (365–375 nm) light emitted by LED lamps (customized at Aijia Electronic Technology). All LED lamps of different wavelengths were standardized to 25 W, with photon densities corresponding to 1.01–1.66 × 10^16^ photons/cm^2^/s, as measured by an HR6 spectrometer (OceanOptics). One arm of the Y‐maze was equipped with a light source while the other was blocked off by a partition and covered with a plastic plate to maintain darkness. A camera was suspended 1.5 meters above the main channel of the Y‐maze to record the movement of the sea cucumbers. During this experiment, 20 sea cucumbers were randomly selected and placed at the terminal of the maze, with their movement observed by the camera. After an hour, their final positions were recorded. The experiment was conducted three times for each light source, with each sea cucumber returned to the culture pond after analysis without reusing them.

### 4.3 Light‐Sensitive Areas of the Sea Cucumber Body, Sensors, and Their Response Spectra

The light‐sensitive areas of the sea cucumber body and their response spectra were determined based on the contraction behavior elicited by illumination. Sea cucumbers were placed in transparent glass tanks under dark conditions, and their anterior end (oral tentacles), dorsal surface, and posterior end (anus) were exposed to white, red, green, blue, and ultraviolet light emitted by LED lamps as described above. This experiment involved 150 individual sea cucumbers, which were divided into 5 groups as biological replicates, with the behavior of 30 individuals being measured in each group for each light source. The contraction response was measured by calculating the number of animals exhibiting a contraction and expressed as a percentage, and the sea cucumbers were returned to the culture ponds and not utilized for at least 24 h following the experiment.

The light sensors on the oral tentacles, papillae, and tube feet of sea cucumbers were identified using red (650 nm), green (532 nm), and blue (450 nm) light produced by laser pointers (Laserland, 11061137, 11051047, and 11040037). All laser pointers of different wavelengths were set to 5 mW (∼1.6 × 10^3^ W m^−2^). If an organ contracted upon illumination by a laser, it was deemed to be responsive to that specific light wavelength. The contraction responses of the organs were captured using a video recorder. This experiment involved 10 individual sea cucumbers as biological replicates, and the experiment was conducted 10 times for each individual with each light source.

### 4.4 Genome‐Wide Identification of Opsins

The proteomes of *H. leucospilota* [43] and 21 other deuterostome species (Table S9), including *Homo sapiens*, *Gallus gallus*, *Xenopus tropicalis*, *Lepisosteus oculatus*, *Ciona intestinalis, Branchiostoma lanceolatum, Saccoglossus kowalevskii, Ptychodera flava, Anneissia japonica, Amphiura filiformis, Acanthaster planci, Asterias rubens, Patiria miniata, Strongylocentrotus purpuratus, Lytechinus variegatus, Lytechinus pictus, Chiridota heheva, Apostichopus japonicus, Stichopus monotuberculatus, and Holothuria scabra*, were selected to assign functions using the SwissProt [85] and InterProscan databases [86]. Opsin‐like genes from these species were identified based on the keyword “opsin” in SwissProt and the keyword “IPR000276, G protein‐coupled receptor, rhodopsin‐like” in InterProscan. Opsins predicted to encode fewer than 5 transmembrane regions were excluded.

### 4.5 Genome‐Wide Identification of Genes Related to the Visual Cycle, Opsin Signaling and Eye Development

Based on the annotations of SwissProt, genes associated with the visual cycle, opsin signaling, and eye development were identified from the *H. leucospilota* genome. Visual cycle‐related genes included retinal pigment epithelium 65 kDa protein like gene (*RPE65*‐like), retinol‐binding protein (*RBP*), lecithin retinol acyltransferase (*LRAT*), and retinol dehydrogenase (*RDH*). Opsin signaling‐related genes included Guanine nucleotide‐binding protein (G‐protein) subunits Gq (*GNAQ*), Gβ (*GNB*), Gγ (*GNG*), 1‐phosphatidylinositol 4,5‐bisphosphate phosphodiesterase‐β (*PLCB*), inactive phospholipase C‐like protein (*PLCL*), and protein kinase C (*PKC*). Eye development‐related genes included paired box protein‐6 (*PAX6*), paired box protein‐2/5/8 (*PAX2*/*5*/*8*), homeobox protein‐3/6 (*SIX3*/*6*), transcription factor SOX‐2 (*SOX2*), bone morphogenetic protein‐2/4 (*BMP2*/*4*), pituitary homeobox‐3 (*PITX3*), neural retina‐specific leucine zipper protein (*NRL*), prospero homeobox protein‐1 (*PROX1*), forkhead box protein‐N4 (*FOXN4*), cone‐rod homeobox (*CRX*), orthodenticle homeobox‐2 (*OTX2*), and retinal homeobox protein (*RAX*).

### 4.6 Phylogenetic Analysis and Classification of Opsins

A phylogenetic analysis was performed with the opsin‐like genes identified from 4 species of Vertebrata (*H. sapiens*, *G. gallus*, *X. tropicalis*, and *L. oculatus*), 1 species of Urochorda (*C. intestinalis*), 1 species of Cephalochordata (*B. lanceolatum*), 2 species of Hemichordata (*S. kowalevskii* and *P. flava*), 4 species of Echinodermata (*A. japonica*, *A. planci*, *S. purpuratus*, and *H. leucospilota*) and 1 species of Placozoa (*Trichoplax adhaerens*). *H. sapiens* melatonin receptors, which are closely related to the rhodopsin‐like G protein‐coupled receptors [87], were chosen as the outgroup. Genes clustering with *H. sapiens* melatonin receptors were first excluded as potential melatonin receptors rather than opsins. Multiple sequence alignments for all gene families mentioned above were built with the MAFFT (v7.487) aligner, and their corresponding phylogenies were inferred with IQ‐TREE (v2.3.6). Phylogenetic trees were constructed to classify gene families and were visualized with Evolview (v3). Specific opsin subtypes were determined by the phylogenetic analysis and annotated according to previous classifications [22, 27, 28, 65].

An additional phylogenetic analysis of opsin was performed using sequences from 13 echinoderm species, including 1 species of class Crinoidea (*A. japonica*), 1 species of class Ophiuroidea (*A. filiformis*), 3 species of class Asteroidea (*A. planci*, *A. rubens*, and *P. miniata*), 3 species of class Echinoidea, and 5 species of class Holothuroidea (*C. heheva*, *A. japonicus*, *S. monotuberculatus*, *H. scabra*, and *H. leucospilota*), with opsin classification determined as described above. Since no opsin gene has been found in *A. japonica*, the final phylogenetic tree includes only opsins from the 12 species.

### 4.7 Identification of Potentially Functional Opsins With a Conserved Chromophore Binding Residue

To identify potentially functional opsins containing the conserved residue K296 (referring to the homologous positions in bovine opsin), which is critical for chromophore binding, protein sequences were identified based on the keyword “IPR027430, Visual pigments (opsins) retinal binding site” in InterProscan. For the obtained proteins, a phylogenetic analysis was conducted as described above, together with sequence alignment and visualization using MEGA (v 7.0.14). The opsin protein sequence alignment was then inspected to identify sequences with the conserved chromophore binding site, along with the NPXXY motif crucial for G‐protein activation, and the tripeptide NxQ and HxK motifs characteristic of the c‐ and r‐opsins, respectively.

### 4.8 RNA Sequencing and Gene Expression Analyses

For RNA sequencing, tissues selected for analysis included the respiratory tree, Cuvierian organ, intestine, rete mirabile, transverse vessel, Polian vesicles, coelomocytes, ovaries, testes, muscle, body wall, and potential photosensitive sites that included the oral tentacles, anus, papillae, and tube feet. In addition, embryonic and larval developmental stages, including fertilized eggs, 2‐cells, 4‐cells, 8‐cells, 16‐cells, morula, blastula, rotated‐blastula, early‐gastrula, late‐gastrula, early‐auricularia, mid‐auricularia, auricularia, doliolaria, pentactula, 1‐mm juvenile, and 20‐mm juvenile, were sampled as previously described [88]. RNA library preparations were sequenced on an Illumina HiSeq platform, generating 150 bp paired‐end reads. Clean data were obtained using SOAPnuke (v1.5.6). Paired‐end clean reads were aligned to the reference genome with HISAT2 (v2.1.0). Transcripts were assembled and read counts for each gene were calculated using StringTie (v1.3.5). Counts per million mapped reads (CPM) were calculated, and cross‐sample normalization was performed using DESeq2 (v1.46.0). Gene expression was visualized with heat maps generated by TBtools (v2.0) software.

### 4.9 RT‐PCR for Opsin Expression

The expression of opsin genes (Hl‐37833, Hl‐07298, Hl‐37282, and Hl‐37244) in photosensitive sites such as the oral tentacles, anus, papillae, and tube feet was further examined using RT‐PCR. Total RNA was isolated using the RNA Easy Fast Tissue/Cell Kit (TIANGEN, DP451) and reverse transcribed into cDNA using the PrimeScript II 1st Strand cDNA Synthesis Kit (TaKaRa, 6210A). RT‐PCR was performed with primers specific for Hl‐37833, Hl‐07298, Hl‐37282, and Hl‐37244 (Table S8) under the following conditions: 34 cycles of 30 s at 94°C, 30 s at 57°C, and 60 s at 72°C. The resulting PCR products were resolved by electrophoresis on a 2% agarose gel and visualized with a GelDoc XR system (Bio‐Rad). In a parallel experiment, RT‐PCR targeting elongation factor 1‐α (Hl‐40064) was conducted as an internal control.

### 4.10 Functional Analysis of R‐Opsin

The coding sequence of r‐opsin (Hl‐37833) from *H. leucospilota* was synthesized with codons optimized for human codon usage. Following truncation at site 359 based on the bovine rhodopsin sequence, the gene was ligated into the pcDNA3.1(+) vector (Invitrogen, V79020), with an in‐frame epitope tag at the C‐terminal end (5’‐ACA GAG ACC AGC CAA GTG GCG CCT GCC‐3’). The plasmid was then transfected into HEK293T cells using EZ Trans (Life iLab, AC04L011). Cells were collected 48 h after transfection and then incubated together with the chromophore (11‐*cis*‐retinal) to regenerate the visual pigment in vitro. After visual pigment regeneration, opsin was purified using the Rho1D4 monoclonal antibody (The University of British Columbia) following published protocols [89, 90]. Finally, the spectral sensitivity (λ_max_) of the purified r‐opsin was measured with a Cary 4000 UV–vis spectrophotometer (Agilent) in the dark, and then measured again after a 60 min blue light exposure (40 W LED lamp, 440–450 nm) at 10°C either in the presence or absence of 100 mm hydroxylamine.

### 4.11 Structural Modeling and Molecular Docking

*H. leucospilota* r‐opsin (Hl‐37833), which was truncated at position 359, was used for in silico molecular binding analysis between the opsin and its chromophore (11‐*cis*‐retinal). We first inferred a 3D structure for the r‐opsin using ColabFold [91], with a total of five models generated. Within them, the model with the highest pLDDT score was selected. Both the modeled r‐opsin structure and the 11‐*cis*‐retinal ligand were converted to PDBQT format following the addition of hydrogen atoms. Covalent docking was then performed using AutoDockFR (v1.0) [92]. The docking search space was defined based on the conserved lysine residue (K331 in Hl‐37833) located in the seventh transmembrane helix. A cubic docking box with dimensions of 18 Å per side was centered on this atom to encompass the predicted ligand‐binding pocket. Finally, the interaction between r‐opsin and the chromophore was visualized using PyMOL (v3.0).

### 4.12 IP_1_ Assay on the R‐Opsin (Hl‐37833)

The IP_1_ assay was performed to determine whether light activation of *H. leucospilota* r‐opsin induces downstream Gq signal transduction, following previously established protocols [93]. HEK293T cells were transfected with *H. leucospilota* r‐opsin (Hl‐37833) and cultured for 48 h. Cells were then harvested and incubated with 11‐*cis*‐retinal to allow regeneration of visual pigment, followed by resuspension in buffer containing LiCl. The suspended cells were transferred to a 96‐well low‐volume plate (Cisbio, 66PL96025). Cells were exposed to blue light (440–450 nm) using a 40 W LED lamp for 3 min and subsequently incubated at 37°C for 1 h. IP_1_ accumulation was quantified using the HTRF IP‐One Gq assay kit (Cisbio, 62IPAPEB) and measured on a VICTOR Nivo multimode microplate reader (Revvity). IP_1_ concentrations were calculated from the fluorescence intensity ratio at 665 and 620 nm. As controls, cells transfected with the blank pcDNA3.1(+) vector and cells expressing *H. leucospilota* r‐opsin without light exposure (dark condition) were processed in parallel following the same experimental procedure.

### 4.13 RNA Interference

Using gene‐specific primers that were designed based on the target sequences (Table S8), double‐stranded RNA (dsRNA) targeting *H. leucospilota* r‐opsin (Hl‐37833) and EGFP (as a non‐targeting negative control) was synthesized using the T7 RiboMAX Express RNAi System kit (Promega, P1700) and diluted in RNase‐free saline solution (RFSS). For the in vivo RNAi experiment, 40 sea cucumbers (approximately 50 g each) were randomly divided into 4 groups of 10 individuals each, and the experiment was conducted as reported before [94]. One group served as a blank control and received no injection. The two negative control groups were injected with 100 µL of EGFP dsRNA solution or RFSS alone, respectively. The experimental group was injected with 100 µL of r‐opsin (Hl‐37833) dsRNA solution. At 24 h post‐injection, the oral tentacles were photostimulated with blue (450 nm), green (532 nm), and red (650 nm) laser pointers, and contraction responses were observed and recorded. This experiment involved 10 individual sea cucumbers as biological replicates, and the experiment was conducted 10 times for each individual with each light source.

### 4.14 Histology

After *H. leucospilota* were anesthetized and relaxed by immersion in a seawater solution containing 5% MgCl_2_, oral tentacle, papillae, and tube feet tissue were collected. These tissues were embedded in paraffin, longitudinally sectioned into 4 µm slices, and then stained with hematoxylin and eosin (H/E). The H/E sections were subsequently observed and imaged using a CaseViewer 2.4 Microscope Slide Scanner (3DHISTECH).

### 4.15 Fluorescence In Situ Hybridization and Immunofluorescence Staining

For fluorescence in situ hybridization (F*IS*H), paraffin sections were deparaffinized and subjected to antigen retrieval in citrate‐EDTA buffer (pH 6.0) at 95°C for 10 min, followed by incubation with 3% H_2_O_2_ at room temperature for 15 min protected from light. Digoxin (DIG)‐labeled antisense cRNA probes for *H. leucospilota* r‐opsin (Hl‐37833) and the *RPE65*‐like gene (Hl‐38728) were generated using a DIG RNA labeling mixture (Roche, 11277073910) and diluted 1:100 in PBS. After pre‐hybridization at 37°C for 1 h, the tissue sections were hybridized with the probe overnight at 42°C, followed by washing with 2× SSC at 37°C for 10 min, 1× SSC at 37°C twice for 5 min each, and 0.5× SSC at room temperature for 10 min to remove unbound probes. The sections were then incubated with biotin‐conjugated AffiniPure mouse anti‐DIG IgG (Boster Bio, BM0040) diluted 1:50 in PBS for 1 h. Subsequently, HRP‐conjugated streptavidin (Invitrogen, ENN100), diluted 1:100 in PBS, was then applied dropwise and incubated for 15 min to amplify the signal. Signal development for Hl‐37833 in the oral tentacles, papillae, and tube feet was performed using the Alexa Fluor 555 Tyramide SuperBoost Kit (Invitrogen, B40933). For the colocalization of Hl‐37833 and Hl‐38728, the Alexa Fluor 488 Tyramide SuperBoost Kit (Invitrogen, B40932) was employed. Cell nuclei were counterstained with DAPI reagent (Roche, 10236276001). The F*IS*H sections were observed and captured using an Eclipse 80i Upright Microscope (Nikon).

For immunofluorescence staining of the nerve fibers, paraffin sections were deparaffinized and subjected to antigen retrieval in citrate‐EDTA buffer (pH 6.0) by bringing the buffer to a boil for 8 min, maintaining it at sub‐boiling temperature for an additional 8 min, and then allowing it to cool for 7 min. Subsequently, the sections were incubated with 3% H_2_O_2_ at room temperature for 25 min protected from light. After blocking with 3% BSA at room temperature for 30 min, the tissue sections were incubated overnight at 37°C with a monoclonal mouse anti‐*β*‐tubulin antibody (Sigma–Aldrich, T5201) diluted 1:200 in PBS. After washing three times with PBS at room temperature, the sections were incubated with a Goat Anti‐Mouse IgG H&L (Cy3) preabsorbed secondary antibody (Abcam, ab97035) for signal development. DAPI reagent was used for counterstaining the cell nuclei. Fluorescence images were observed and acquired using an Eclipse 80i Microscope (Nikon).

### 4.16 Electron Microscopy Analysis

Oral tentacle, papillae, and tube feet samples were fixed in 2.5% glutaraldehyde at room temperature for 2 h, and then transferred to 4°C. For ultrastructural analysis by scanning electron microscopy (SEM), the samples were rinsed several times in PBS and post‐fixed in 1% osmium tetroxide for 2 h. The samples were then dehydrated in a graded ethanol series, transferred to isopentyl acetate, and dried using a K850 critical point dryer (Quorum, East Sussex). After mounting on stubs, samples were sputter‐coated with gold using MC1000 Ion Sputter Coater (Hitachi), attached to metallic stubs, and visualized with an SU8100 scanning electron microscope (Hitachi).

For ultrastructural analysis by transmission electron microscopy (TEM), the fixed samples were dehydrated in a graded ethanol series and then permeabilized using a mixture of acetone and epoxy resin (2:1). Samples were sectioned into 60 nm thin slices using an EM UC7 Ultramicrotome (Leica). The sections were stained with saturated aqueous uranyl acetate solution and lead citrate solution, each for 15 min at room temperature, followed by overnight drying at room temperature. The grids were subsequently visualized with a JEM‐1400Flash transmission electron microscope (JEOL).

### 4.17 Statistics Analysis

Statistical analysis was performed using GraphPad Prism 10.0 (GraphPad Software). All data are presented as the mean ± standard error of the mean (SEM) of 3–10 independent experiments. Statistical differences were estimated via one‐way ANOVA followed by Tukey's multiple comparisons test, where n.s. *p* ≥ 0.05, ^*^ *p* < 0.05, ^**^ *p* < 0.01, ^***^ *p* < 0.001, and ^****^ *p* < 0.0001.

## Author Contributions

**Y.Z., C.H., Y.L. (2), and T.C**. conceptualized the study; **J.H., Z.Q., Y.C., B.Z., X.W., Y.X., X.Z., A.Y., Y.L (1), and W.P**. performed research; **C.R., P.L., A.Y., X.J., L.Y., H.Q., C.H., Y.L. (2), and T.C**. contributed samples/reagents/analytic tools; **J.H., C.R., Z.Q., Y.C., B.Z., P.L., A.Y., D.F., Y.L. (2), and T.C**. analyzed data; **J.H., C.R., P.L., X.W., D.M.I., C.H., Y.L. (2), and T.C**. wrote the paper. All authors have read and approved the submitted version of the manuscript.

## Funding

This study was supported by grants from the Guangdong Province Project (2024A1515011418 and 2023B1212060047), the National Natural Science Foundation of China (32270462, 42176132, and 32573487), the Science and Technology Program of Nansha District (NSJL202103), the National Key R & D Program of China (2022YFD2401301), and the Research on breeding technology of candidate species for Guangdong modern marine ranching (2025‐MRB‐00‐001).

## Conflicts of Interest

The authors declare no conflicts of interest.

## Supporting information




**Supporting File 1**: advs76743‐sup‐0001‐SuppMat.docx.

**Supporting File 2**: advs76743‐sup‐0002‐FigureS1‐S15.docx.

**Supporting File 3**: advs76743‐sup‐0003‐TableS1‐S10.xlsx.

**Supporting File 4**: advs76743‐sup‐0004‐VideoS1‐S4.zip.

## Data Availability

RNA‐seq data from the various tissues, photosensitive‐sites and developmental stages have been deposited into the GenBank SRA database under the accession nos. SRR15275174–SRR15275209 and SRR30574884–SRR30574903 (Table S10), respectively. All data are available in the main text or the supplementary materials.
