# Supplementary material for: A Pentaradial and Bilateral Combined Photoreception System in the Sea Cucumber is Mediated by a Blue Light‐Sensitive R‐Opsin
Source: Adv Sci (Weinh). 2026 Jul 27:e12095. Online ahead of print. doi: 10.1002/advs.202512095 (PMC13403383; doi:10.1002/advs.202512095)
Supplement: Supplementary file 1 — Supporting File 1: advs76743‐sup‐0001‐SuppMat.docx. [file ADVS-9999-e12095-s002.docx]

**Supplementary Figures**

**Figure S1.** A detailed maximum likelihood (ML) phylogenetic tree of opsins and opsin-like genes in 12 typical Deuterostomia (*H. sapiens*, *G. gallus*, *X. tropicalis*, *L. oculatus*, *C. intestinalis*, *B. lanceolatum*, *S. kowalevskii*, *P. flava*, *A. planci*, *A. japonica*, *S. purpuratus*, and *H. leucospilota*) and a Placozoa (*T. adhaerens*) species. Red, blue, green, and purple represent the sequences from species of vertebrates, echinoderms, non-echinoderm deuterostome invertebrates, and placozoans, respectively. Additionally, the opsin lineages are marked with lines of different colors, and the confidence of the nodes is also indicated by numbers.

**Figure S2.** Conserved sequence characteristics of the seventh transmembrane domain of Deuterostomia opsins containing retinal binding sites.

**Figure S3.** RT-PCR confirmation of opsin mRNA expression in photosensitive sites including the oral tentacles, papillae, and tube feet.

**Figure S4.** Details for the heatmap illustrating transcript expression levels of genes related to the visual cycle and opsin signaling in different tissues and photosensitive-sites, as described above. *RPE65*-like, retinal pigment epithelium 65 kDa protein like; *RBP*2, retinol-binding protein 2; *LRAT*, lecithin retinol acyltransferase; *RDH*, retinol dehydrogenase; *GNAQ*, G protein subunit alpha q; *GNB*, G protein subunit beta; *GNG*, G protein subunit gamma; *PLCB*, 1-phosphatidylinositol 4,5-bisphosphate phosphodiesterase beta; *PLCL*, inactive phospholipase C-like protein; *PKC*, protein kinase C.

**Figure S5.** Spectral tuning of sea cucumber r-opsin. The black and red lines represent the absorption spectra in the dark state and after light exposure (without hydroxylamine), respectively. The blue line in the inset shows the dark minus light difference spectrum.

**Figure S6.** Biological and technical repeats for IP_1_ assay for H. leucospilota r-opsin (Hl-37833) expressed in cultured cells.

**Figure S7.** Efficiencies of RNAi against r-opsin (Hl-37833) in the oral tentacles of *H. leucospilota* after injection of RFSS (blank control) or dsRNA of EGFP (negative control), r-opsin (Hl-37833) for 12 h. Data presented here are expressed as mean±SEM (n=10 from 10 individuals; *P* values are calculated by one-way ANOVA followed by Tukey’s multiple comparisons test, where * *P* < 0.05, ** *P* < 0.01, *** *P* < 0.001, **** *P* < 0.001).

**Figure S8.** A detailed maximum likelihood (ML) phylogenetic tree of opsins and opsin-like genes from 12 echinoderm species (*A. filiformis*, *A. planci*, *A. rubens*, *P. miniata*, *S. purpuratus*, *L. variegatus*, *L. pictus*, *C. heheva*, *A. japonicus*, *S. monotuberculatus*, *H. scabra*, and *H. leucospilota*). Red, blue, green, and purple represent sequences from species of starfish, sea cucumber, sea urchin, and brittle stars, respectively, with opsins containing the retinal binding site indicated by a star. Additionally, the opsin lineages are marked with lines of different colors, and the confidence of the nodes is also indicated by numbers.

**Figure S9.** Morphology and histology of the photosensitive oral tentacles, papillae, and tube feet in *Holothuria leucospilota*.

**Figure S10.** High-resolution images of H&E staining showing the histology of the photosensitive oral tentacles, papillae, and tube feet. PT, petiole tentacles; ST, scutiform tentacles; Di, disc; Su, sucker; EpL, epithelial layer; LCT, loose connective tissue; DCT, dense connective tissue; NL, nerve layer; ML, muscle layer; BCL, body cavity lining; WVC, water vascular cavity.

**Figure S11.** High-resolution and negative-control images from F*IS*H showing r-opsin (Hl-37833) mRNA expression in the photosensitive oral tentacles, papillae, and tube feet. P, papillae; B, buds.

**Figure S12.** High-resolution and high-magnification SEM for internal ultrastructures of photosensitive oral tentacles, papillae, and tube feet.

**Figure S13.** High-resolution TEM images for the internal ultrastructure of the photosensitive oral tentacles, papillae, and tube feet.

**Figure S14.** High-resolution and negative-control images for anti-β-tubulin staining of nerve bundles (red), with cell nuclei are stained with DAPI (blue) in the photosensitive oral tentacles, papillae, and tube feet. EN, epineural nerve plexus; HN, hyponeural nerve plexus.

**Figure S15.** High-resolution and negative-control images for F*IS*H showing the colocalization of *RPE65*-like gene (Hl-38728) and r-opsin (Hl-37833) mRNA expression in the photosensitive oral tentacles. B, buds; WVC, water vascular cavity.

**Supplementary Tables**

**Table S1.** Opsins in *H. leucospilota* and 20 other deuterostome species.

**Table S2.** Transcript levels of opsins, and genes related to the visual cycle, opsin signaling and eye development in multiple tissues of *H. leucospilota*, as represented by CPM. The selected tissues include respiratory tree (RT), Cuvierian organ (CO), intestine (In), rete mirabile (RM), transverse vessel (TV), Polian vesicle (PV), ovary (Ov), testis (Ts), muscle (Ms), and body wall (BW).

**Table S3.** Transcript levels of opsins, and genes related to the visual cycle, opsin signaling and eye development in multiple photosensitive sites of *H. leucospilota*, as represented by CPM. The selected tissues include oral tentacles (OT), anus (An), papillae (Pp), and tube feet (TF).

**Table S4.** Spectral tuning of r-opsin Hl-37833.

**Table S5.** Transcript levels of opsins in tissues of other 3 holothurian species (*A. japonicus*, *S. monotuberculatus*, and *H. scabra*), as represented by CPM. The selected tissues include oral tentacles (OT), body wall (BW), muscle (Ms), rete mirabile (RM), respiratory tree (RT), and Polian vesicle (PV).

**Table S6.** Transcript levels of opsins, and genes related to the visual cycle, opsin signaling and eye development in different embryonic and larval developmental stages of *H. leucospilota*, as represented by CPM. The selected stages include fertilized eggs (FE), 2-cells (2C), 4-cells (4C), 8-cells (8C), 16-cells (16C), morula (Mr), blastula (Bs), rotated-blastula (RB), early-gastrula (EG), late-gastrula (LG), early-auricularia (EA), mid-auricularia (MA), auricularia (Ar), doliolaria (Dl), pentactula (Pt), 1-mm juveniles (J1), and 20-mm juveniles (J20).

**Table S7.** Genes related to eye development in *H. leucospilota* and 16 other deuterostome species.

**Table S8.** Information regarding the reagents and oligonucleotides used in this study.

**Table S9.** The genome databases used for comparison in this study.

**Table S10.** Sample sequencing information in this study.

**Supplementary videos**

**Video S1.** Phototaxis of *H. leucospilota* to white, red, green, blue, and UV light.

**Video S2.** Contraction responses of *H. leucospilota* photosensitive sites to red laser pointer irradiation.

**Video S3.** Contraction responses of *H. leucospilota* photosensitive sites to blue laser pointer irradiation.

**Video S4.** Contraction responses of *H. leucospilota* photosensitive sites to green laser pointer irradiation.
